# DCF intraperitoneal and intravenous dual chemotherapy regimen for advanced gastric cancer: A feasibility study

**DOI:** 10.3892/ol.2014.2651

**Published:** 2014-10-31

**Authors:** ZENG-LI FENG, LIU-BIN CHEN, ZHEN-YU LIU, XUE-JI CHEN, XIAO-CAN REN, YUE-E LIU, YU PENG, HAI-GANG WANG, SHUN-MAO MA, FENG-JIE MENG, QIANG LIN

**Affiliations:** 1 Department of General Surgery, North China Petroleum Bureau General Hospital of Hebei Medical University, Renqiu, Hebei 062552, P.R. China; 2 Department of Ultrasound Radiology, North China Petroleum Bureau Youjian Hospital, Renqiu, Hebei 062552, P.R. China; 3 Department of Surgery, North China Petroleum Bureau Caiyi Hospital, Renqiu, Hebei 062552, P.R. China; 4 Department of Oncology, North China Petroleum Bureau General Hospital of Hebei Medical University, Renqiu, Hebei 062552, P.R. China

**Keywords:** gastric cancer, intraperitoneal chemotherapy, dual chemotherapy, docetaxel, cisplatin, fluorouracil

## Abstract

Gastric cancer is the fourth most common type of cancer globally and accounts for the second highest cancer-associated mortality rate in the world. Current treatment strategies for gastric cancer include surgery, radiotherapy, chemotherapy and targeted therapy. Intraperitoneal (IP) chemotherapy may increase the IP concentrations of chemotherapy drugs and reduce the systemic toxicity. At present, IP chemotherapy is used to treat patients with advanced gastric cancer, which has a high rate of peritoneal recurrence. The present study evaluated the feasibility of using docetaxel, cisplatin and fluorouracil (DCF) in an IP and intravenous (IV) dual chemotherapy regimen for the treatment of advanced gastric cancer. The treatment-associated adverse reactions and preliminary efficacy were reported. The first dose level utilized the full dose of DCF: Docetaxel, day one, 45 mg/m2 (IP) and day eight, 30 mg/m2 (IV); cisplatin (DDP), day one, 75 mg/m2 (IP); and fluorouracil (FU), days one to five, 750 mg/m2 (continuous IV). A total of six patients were treated at this level and two patients withdrew due to serious adverse reactions. Taking into account that the the tolerated doses used in combination regimens for Eastern populations are lower than that of the corresponding doses for Western populations, the dosages of the three drugs were all reduced by 20% in the application of the second dose level: Docetaxel, day one, 30 mg/m2 (IP) and day eight, 30 mg/m2 (IV); DDP, day two, 60 mg/m2 (IP); and FU, days one to five, 600 mg/m2 (continuous IV). A total of 26 patients were treated at this level. The main adverse reaction was bone marrow suppression, with grade III/IV neutropenia, leukopenia and febrile neutropenia accounting for 61.5, 53.8 and 19.2% of reactions, respectively, and grade III/IV anemia and thrombocytopenia accounting for 19.2 and 15.4% of reactions, respectively. Gastrointestinal adverse reactions primarily consisted of abdominal pain, with grade III/IV abdominal pain accounting for 30.8% of reactions. Only 7.7% of the patients withdrew from the treatment. The median time to progression (TTP) was five months [95% confidence interval (CI), 1.0–9.0 months], and the median overall survival (OS) was nine months (95% CI, 7.4–10.6 months). It was concluded that the DCF regimen with reduced dosage should be applied. IP and IV dual chemotherapy for the treatment of unresectable advanced gastric cancer is tolerated and demonstrated a good initial efficacy. Strategies for mitigating and reducing the adverse gastrointestinal reactions, particularly abdominal pain, may be the focus of future studies.

## Introduction

According to global cancer statistics, gastric cancer is the fourth most common cancer and exhibits the second highest mortality rate. China possesses a high incidence of gastric cancer (1). Due to its atypical symptoms, gastric cancer is often locally advanced or has undergone distant metastasis at the time of clinical diagnosis, leading to a poor prognosis. Even with radical surgery, the five-year survival rates for stage III and IV gastric cancers are only 28.0 and 18.4%, respectively (2). Although there is no globally accepted standard regimen for the treatment of gastric cancer, systemic chemotherapy is superior to the best supportive care (3,4).

Studies have revealed that the local recurrence rate for locally advanced gastric cancer remains as high as 30.4%, even subsequent to D2 radical surgery. The peritoneum is the first site of recurrence, accounting for up to 58.8% of recurrences. Therefore, it has been recommended that, for postoperative adjuvant therapy, intraperitoneal (IP) chemotherapy should be considered (5). The extent of the local regional spread is an important component of the natural history of gastric cancer. Thus, even if there is no clinical evidence of peritoneal dissemination, strong reasons for local-regional treatment remain. IP chemotherapy has become increasingly popular for use in clinical practice and has achieved a certain amount of success. However, no standards exist regarding drug choices and administration approaches (6).

V325, the phase III randomized controlled trial, confirmed the significance of docetaxel in treating advanced gastric cancer, and the overall response rate (ORR), time to progression (TTP) and overall survival (OS) of the docetaxel, cisplatin (DDP) and fluorouracil (FU) (DCF) regimen were significantly improved compared to the DDP and FU (CF) regimen (7). A previous meta-analysis confirmed the superiority of docetaxel in treating gastric cancer (8). Notably, the DCF regimen has been reported to exhibit strong toxicity, with 69% of patients experiencing treatment-associated grade III/IV adverse reactions, and the rate of grade III/IV neutropenia reaching 82%, while that of febrile and infectious neutropenia was 29%. Such serious adverse reactions affect the application of the DCF regimen. In addition to increasing the IP concentrations of chemotherapy drugs, IP chemotherapy can also reduce the systemic toxicity (5). Additionally, the unique pharmacokinetic characteristics of docetaxel are suitable for application via IP perfusion (9,10). Theoretically, a DCF IP and intravenous (IV) dual chemotherapy regimen should be able to achieve a good treatment effect and reduce systemic toxicity.

Studies using IP and IV dual chemotherapy as the sole means of treatment for advanced gastric cancer are rare. To the best of our knowledge, the present study is the first study to report DCF dual IV and IP chemotherapy.

## Materials and methods

### Eligibility

The present study enrolled patients from North China Petroleum Bureau General Hospital of Hebei Medical University (Renqui, China) in the III–IV clinical stages of disease with unresectable gastric cancer that was pathologically or cytologically confirmed, locally-advanced, metastasized or recurrent, and who possessed at least one evaluable lesion. The inclusion criteria were as follows: 18–75 years old; Karnofsky performance status (KPS) score ≥60 and expected survival of at least three months; complete recovery from the toxicity of previous treatment; and a period of at least four weeks since the previous treatment. The bone marrow conditions were as follows: White blood cell (WBC) count, ≥4.0 × 10^9^/l; neutrophil count, ≥2.0 × 10^9^/l; platelet (PLT) count, ≥100 ×10^9^/l; and hemoglobin level, ≥100 g/l. The required blood creatinine level was ≤ 135 μmol/l, the alkaline phosphatase level was required to be <1.5 times the upper limit of the normal level and the required serum bilirubin level was ≤26 μmol/l. Other inclusion criteria consisted of no significant gastrointestinal bleeding, normal heart and lung function, no significant systemic infection and no other serious visceral disease, no previous application of docetaxel, good dependability, willing and able to comply with the regimen during the study period and provided written informed consent.

### Exclusion criteria

The following patients were excluded: Pregnant or lactating women; patients with no consciousness or with uncontrollable central nervous system metastasis and uncontrollable seizures or who lost consciousness or judgment due to psychosis; patients who had received other chemotherapy drugs or radiation therapy over the past four weeks; patients with organ transplantation; and patients with long-term use of immunosuppressive agents and adrenocortical hormones.

### Withdrawal criteria

The withdrawal criteria consisted of patients who asked to withdraw; patients who had drug allergies or experienced serious adverse reactions or events in the trial and; those who experienced disease progression in the trial.

### Assessment prior to treatment

Medical history, physical examination, KPS score evaluation, a routine blood test, liver function and kidney function tests, chest and abdominal computed tomography (CT) and electrocardiography (ECG) were completed one week prior to treatment.

### Trial design

The present study was a prospective, open-label, single-arm feasibility study. The main endpoint of this study was to evaluate the tolerability of dual IP and IV DCF chemotherapy in advanced gastric cancer. The secondary endpoint was to obtain the ORR, TTP and OS of the chemotherapy.

### Ethics

The procedures were approved by the Ethics Committee of Hebei Medical University (Shijiazhuang, Hebei, China) and were performed in accordance with the ethics standards of human experimentation and with the Helsinki Declaration of 1975, as revised in 2000. The patients provided written informed consent.

### Chemotherapy

#### IP chemotherapy

A peritoneal catheter was implanted and, in the presence of ascites, drainage was performed to remove as much of the ascites as possible. A total of 1,000 ml normal saline, 10 mg dexamethasone and 20 ml 5% lidocaine were injected through the catheter, and the chemotherapy drugs were then injected. Based on the peritoneal conditions of the patient, 1,000–1,500 ml normal saline was injected again. All perfusion liquids were at room temperature. Following the perfusion, the patients were asked to sequentially take right lateral decubitus, left lateral decubitus, prone and supine positions, each for 15 minutes, to allow the drugs to be distributed in the abdominal cavity as uniformly as possible.

#### First dose level (Level I)

Level I consisted of the full-dose DCF regimen: Docetaxel at 40 mg/m^2^ via IP perfusion on day one and 35 mg/m^2^ via IV infusion on day eight; DDP (DDP) at 75 mg/m^2^ via IP perfusion on day two; FU (FU) at 750 mg/m^2^ via continuous IV infusion once per day for five days.

#### Second dose level (Level II)

The Level II DCF regimen consisted of a reduced dosage, as the first six patients experienced intolerable adverse reactions. It was reasoned that the full dose of the DCF regimen resulted in excessive toxicity. Thus, from the seventh patient onward, the chemotherapy dosages of all three drugs were reduced by 20%. This provided the doses of docetaxel at 30 mg/m^2^ via IP perfusion on day one and 30 mg/m^2^ via IV infusion on day eight; DDP at 60 mg/m^2^ via IP perfusion on day two; and FU at 600 mg/m^2^ via continuous IV infusion once per day for five days.

Prophylactic anti-allergy treatment was applied with 10 mg of dexamethasone twice per day one day prior to treatment and on days one and two, for three consecutive days. The treatment was repeated every 28 days until disease progression or the occurrence of intolerable toxicity. The maximum number of treatment cycles was six. During the treatment, all patients were given 5-HT3 receptor antagonists for antiemetic prophylactic treatment. To ensure the continuity of chemotherapy, recombinant human granulocyte colony-stimulating factor was administered for supportive treatment when the WBC count was <4.0 × 10^9^/l or the absolute neutrophil count (ANC) was <2.0 × 10^9^/l, and interleukin-11 treatment was applied when PLT was <75 × 10^9^/l. Appropriate supportive treatments, including oral drug administration for the enhancement of WBCs and PLTs, anemia correction and IV rehydration, were applied when indicated.

### Evaluation standards

Assessment of adverse reactions was based on the Common Terminology Criteria for Adverse Events v3.0. RECIST1.1 was used for the evaluation of short-term efficacy (11). Efficacy was evaluated for patients who completed two or more cycles of chemotherapy. The time point for efficacy evaluation was the eighth week after the initiation of the treatment. Efficacy evaluation was divided into complete response (CR), partial response (PR), stable disease (SD) and progressive disease (PD). The response rate (R) was calculated as CR + PR. The main imaging evidence for the evaluation was from CT/magnetic resonance imaging (MRI), and superficial lymph nodes were examined by B-ultrasonography.

### Follow-up

Following completion of the treatment, follow-up studies were conducted once every two months in the first six months and then once every three months, subsequently. Each follow-up study included medical history, physical examination, routine blood tests, comprehensive biochemical examinations, chest and abdominal CT and superficial lymph node B-ultrasonography. All patients were followed up via re-examinations in the outpatient clinic and by telephone, and all patients were followed up until mortality due to any reason or loss of follow-up.

### Statistical analysis

SPSS 19.0 software (IBM, Armonk, NY, USA) was used for data analysis, and the Kaplan-Meier method was used to calculate the TTP and OS of the patients.

## Results

### Patient characteristics

Between July 2010 and June 2013, a total of 32 advanced gastric cancer patients who all possessed unresectable lesions were enrolled in the present study. In total, 59.4% (19/32) of patients possessed ascites and 53.1% (17/32) possessed visceral metastases. There were 19 males and 13 females, with 17 cases receiving treatment for the first time, eight cases being retreated and seven cases being treated for recurrence or metastasis. The age range of the patients was 39–75 years, with a median of 65 years. The median KPS score was 70 (range, 60–90), with seven cases receiving scores of 60, 18 cases receiving scores of 70, six cases receiving scores of 80 and one case receiving a score of 90 (Table I). The body surface area ranged between 1.52–1.89 m^2^, with a median of 1.74 m^2^. There were six stage IIIB cases and 26 stage IV cases. In total, 28 cases exhibited evaluable efficacy, and all 32 cases had evaluable adverse reactions.

As of 10 December 2013, there was loss of follow-up in two cases, resulting in a follow-up rate of 93.8%.

### Completion of treatment

The 32 patients completed a total of 113 cycles of chemotherapy, with a median of four chemotherapy cycles (range, 1–6). Among these patients, four cases completed one cycle, two cases completed two cycles, six cases completed three cycles, 15 cases completed four cycles, one case completed five cycles and four cases completed six cycles.

### Adverse reactions from Level I

Table II describes the hematological toxicity. The six cases treated using Level I developed severe bone marrow suppression. In particular, the incidence rates of grade III/IV neutropenia and leukopenia were 83.3%, while the incidence rate of febrile leukopenia was 33.3%. The rates of grade III/IV anemia and thrombocytopenia were 33.3%. In addition, this group also experienced severe nonhematological toxicity, as shown in Table III. All six patients experienced abdominal pain, and grade III/IV pain was present in 66.7% of cases. Due to their abdominal pain, three patients received opioid analgesics, two of these three cases only completed one cycle of chemotherapy prior to requesting to withdraw from the trial as the pain was not tolerable. The rates for grade III/IV decreased appetite, fatigue, nausea and vomiting were 66.7, 50, 50 and 33.3%, respectively. Therefore, the full dose of the DCF regimen was considered to be too strong for Asian populations. Based on studies from East Asia (12–14) and the results of previous chemotherapy dose studies (15,16), a DCF regimen with a 20% dosage reduction was applied in the subsequent treatment.

### Adverse reactions from Level II

Following a 20% reduction in DCF dosages, the incidence of bone marrow suppression was significantly reduced, and the rates of grade III/IV neutropenia, leukopenia and febrile neutropenia were 61.5, 53.8 and 19.2%, respectively. The rates of grade III/IV anemia and thrombocytopenia were 19.2 and 15.4%, respectively. Although the incidence rate of abdominal pain remained at 100%, the rate for severe grade III/IV pain was 30.8%, and only 7.7% (2/26) of patients terminated the treatment subsequent to the completion of one cycle of chemotherapy due to abdominal pain. Overall, sensory neuropathy was not common, with an incidence of 21.9%, with 6.3% of patients experiencing grade III sensory neuropathy, and no sensory neuropathy cases at grade IV.

### Short-term efficacy

Among the 32 patients, 28 patients were evaluable. There were no CR cases, while there were eight PR cases, 18 SD cases and two PD cases among the 28 patients, providing the RR of 28.6% (8/28).

### Survival analysis

**Figure 1:**
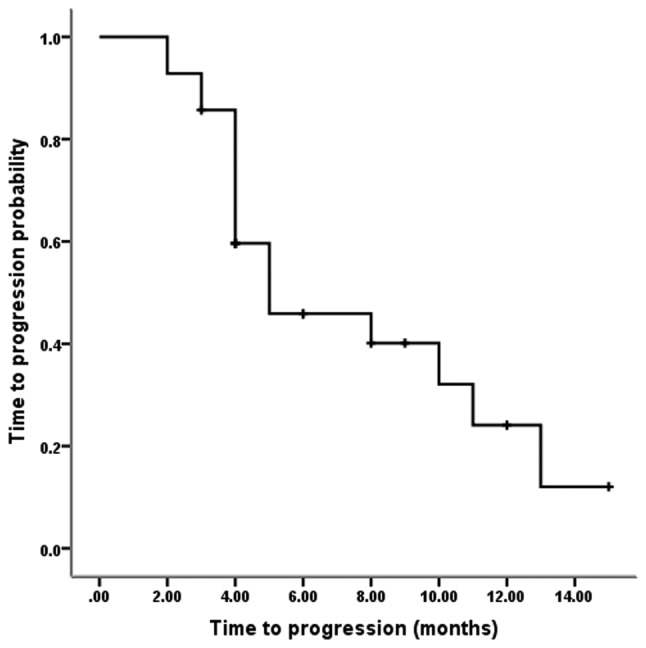
Time to progression (TTP). The median TTP was 5.0 months and the one-year progression rate was 24.1%.

**Figure 2:**
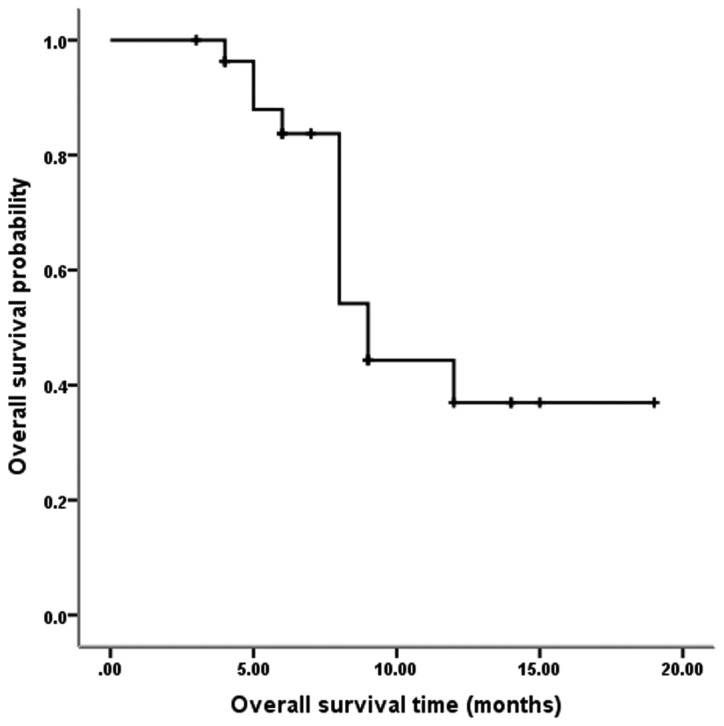
Overall survival (OS). The median OS was 9.0 months and the one-year OS rate was 36.9%.

The median follow-up time of eight months (range, 3–19 months) was relatively short, and the survival data are not yet complete. Nevertheless, the preliminary survival data was reported. Fig. 1 shows that among the 28 evaluable patients, the median TTP was five months (95% CI, 1.0–9.0 months), and the one-year progression rate was 24.1%. Fig. 2 shows that among the 28 evaluable patients, the median OS was nine months (95% CI, 7.4–10.6 months), and the one-year OS was 36.9%.

## Discussion

Chinese gastric cancer patients account for almost half of all gastric cancer patients worldwide (1). Likely due to cultural background and economic reasons, a gastric cancer early screening system has not been implemented in China, and patients are often diagnosed only subsequent to exhibiting apparent clinical symptoms, when cancer staging is often advanced and when radical surgical resection is an option for only a small proportion of patients (17). Chemotherapy plays an important role in advanced gastric cancer, as it has been demonstrated that chemotherapy is superior to the best supportive care. In addition, first- and second-line chemotherapy treatments can improve survival (18,19). However, no standard chemotherapy regimen has been established for gastric cancer (19,20).

Gastric cancer primarily spreads through the blood and peritoneal fluid, with more cases of peritoneal fluid dissemination than blood spread. Among gastric cancer patients, 40% succumb to liver metastases, and 53–60% succumb to peritoneal carcinomatosis (21). Therefore, establishing a protocol to effectively eliminate peritoneal carcinomatosis may become one of the key means of improving treatment efficacy (6).

The use of IP chemotherapy as an adjuvant or neoadjuvant treatment combined with surgery has become a current hot topic (21–27). Post-operative adjuvant IP chemotherapy has shown encouraging efficacy (21–23). However, for patients with extremely advanced gastric cancer, neoadjuvant IP chemotherapy combined with surgery requires a high degree of selectivity. Such treatment not only requires patients to be in a good physical condition and have no significant visceral metastasis, but also requires precise, complex and expensive staging and restaging means, such as one or more laparoscopic examinations (24,25,26), which are difficult to achieve under the current Chinese healthcare resource allocation and economic levels. For patients with advanced gastric cancer, who are in generally poor condition and often possess massive ascites and organ metastases, such strong comprehensive treatment is even more difficult to implement.

V325 performed a well-designed, randomized, multinational phase III trial. V325 enrolled gastric cancer groups with relatively poor prognoses, 97% possessed metastases, 81% possessed metastases involving two or more organs and 57% had experienced a weight loss of >5%. V325 also excluded all patients who could potentially receive surgery. Even for such a group of advanced gastric cancer, docetaxel combined with CF significantly improved the OS and TTP compared with CF. The OS and TTP of docetaxel combined with CF were 5.6 and 9.2 months, and those of CF were 3.7 and 8.6 months, respectively (7). However, DCF resulted in serious treatment-associated adverse events, with ≤82% neutropenia and 29% febrile and infectious neutropenia, which significantly limits DCF application (7). A previous meta-analysis has confirmed that the two-year OS of the combination of three drugs, including docetaxel, was significantly increased compared with regimens without taxanes. However, the same study also reported that the DCF regimen significantly increased the incidence of febrile neutropenia, neutropenia, leukopenia and diarrhea. Therefore, it is crucial to reduce the adverse reactions of the DCF regimen (8).

IP chemotherapy possesses certain advantages compared with systemic chemotherapy. IP chemotherapy is capable of forming high drug concentrations in the peritoneal cavity, reducing systemic toxicity and forming high concentrations in the portal vein. Thus, IP chemotherapy exerts a good treatment effect on liver metastasis, which is one of the main causes of gastric cancer-associated death (28). Docetaxel exhibits unique pharmacokinetic characteristics, making it ideal for IP perfusion. Animal experiments have revealed that the docetaxel concentration in the peritoneal fluid 90 min after IP administration of docetaxel is >2500 times the level achieved by IV administration. The area under the curve (AUC) of peritoneal fluid was 976 times the AUC of the plasma, and the drug concentrations in the abdominal wall, stomach and colon tissue following IP administration were also significantly higher compared with IV administration (9). In human trials, the AUC of peritoneal fluid was 515 times greater compared with the plasma subsequent to IP perfusion of docetaxel at 45 mg/m^2^ (10). Additionally, DDP is one of the most common intraperitoneally administered chemotherapy drugs (6). Therefore, DCF IP and IV dual chemotherapy can theoretically achieve a good effect and result in reduced toxicity compared with IV administration.

As demonstrated by the present exploratory small-sample study, the six patients treated with the first dose level experienced relatively serious bone marrow suppression, with incidence rates ≤83.3% for grade III/IV neutropenia and leukopenia and ≤33.3% for febrile leukopenia. In addition, the incidence rates of grade III/IV anemia and thrombocytopenia also reached 33.3%, and the rates of grade III/IV abdominal pain reached 66.7%. Two cases completed only one cycle of chemotherapy prior to terminating the treatment. The severe adverse reactions may be due to the differences in physical conditions between eastern and western populations. Certain studies from East Asia have reported that Asians exhibit a lower chemotherapy tolerance compared with western populations (12–16). Therefore, the DCF dosage was reduced by 20% and the subsequent study was conducted using this second dosage level. As a result, bone marrow suppression and gastrointestinal symptoms were significantly reduced. The incidence rates of grade III/IV neutropenia, leukopenia and febrile neutropenia were 61.5, 53.8 and 19.2%, respectively. Following supportive treatment, the majority of patients adhered to the treatment. Only two cases terminated the treatment due to abdominal pain following the completion of one cycle of chemotherapy.

Among the total 32 patients, four patients received only one cycle of chemotherapy treatment due to adverse reactions. The median number of chemotherapy cycles completed was four. During the second-stage study using the modified dosage, the hematological toxicity of the DCF IP and IV dual chemotherapy regimen was significantly reduced compared with the V325 study, in which IV administration of DCF was applied (7). However, abdominal pain increased significantly, and grade III/IV pain reached an incidence of 30.8% in the present study. By contrast, the V325 study did not report significant abdominal pain, suggesting that the abdominal pain in the present study was directly associated with IP chemotherapy. Other gastrointestinal symptoms, consisting of anorexia, nausea and vomiting, were comparable to the rates observed in the V325 study, with incidences of 11.5, 15.9 and 7.7% in the present study vs. 10, 14 and 10% in the V325 study, whereas the incidence of diarrhea was reduced in the present study (11.5 vs. 19%).

The incidence of abdominal pain in the present study was significantly higher compared with other studies of docetaxel IP perfusion. One of the studies reported a rate of 18.5%, with pain at grade II or less (24), and three other studies reported that IP treatment did not cause significant pain (25–27). There are three possible reasons for the abdominal pain in the present study. First, the two chemotherapy drugs, docetaxel and DDP, were applied in perfusion, thus increasing abdominal irritation and pain. Secondly, in the IP approach, 2,000–2,500 ml of normal saline at room temperature was infused so that the drugs could be evenly distributed in the abdominal cavity, which increased abdominal bloating and pain. Third, the enrolled patients possessed multi-organ metastasis and poor physical conditions. These patients exhibited relatively poor tolerance for the treatment and may already have experienced symptoms of abdominal pain. However, pain control and other symptomatic treatments were implemented so that the majority of patients could adhere to the chemotherapy regimen.

The present study obtained a TTP of five months and a median survival time (MST) of nine months, which is comparable to the results of the V325 study and similar to the results of a previous meta-analysis (8). However, there appears to be a large gap between the present survival results and those of other docetaxel peritoneal perfusion studies (24–26). A study of 18 cases treated with docetaxel IP chemotherapy plus oral administration of S-1 reported an MST of 24.6 months. The 18 patients enrolled in this study were able to receive radical surgery, and 88.9% (16/18) of them received radical surgery following neoadjuvant therapy (26). The one-year OS rates reported by two other docetaxel IP chemotherapy studies were 70.4 (24) and 78% (25), which are much higher compared with the present rate of 36.9%. The two aforementioned studies mainly enrolled gastric cancer patients who could potentially receive radical surgery, and the patients experienced good physical conditions, those with Eastern Cooperative Oncology Group (ECOG) scores of 0 or 1 (equivalent to KPS scores of 100 and 90) accounted for 100 and 95% of the patient populations in the two studies. The organ metastasis rates in the two studies were 11.1 and 15%, respectively, and subsequent to IP chemotherapy, 51.9 and 40% of the patients, respectively, underwent radical surgery (24,25). By contrast, all patients in the present study possessed unresectable cancer, 59.4% of the patients possessed ascites, 53.1% experienced visceral metastases, the KPS scores of 78.1% of patients were in the range of 60–70, and only 3.1% possessed KPS scores in the range of 90–100. The prognoses of the patients enrolled in the present study were not comparable with the prognoses of the patients enrolled in the aforementioned two studies (24,25).

The current study contained three drawbacks First, due to the small sample size, there may be bias in the present study and it is difficult to conduct subgroup analyses to identify specific populations that are more likely to benefit from DCF dual chemotherapy. Second, as the data collection was not detailed enough, it was not clarified whether the gastrointestinal symptoms were caused by advanced gastric cancer itself or by IP treatment; thus, the abdominal pain, bloating and other adverse reactions associated with IP chemotherapy may have been overestimated. Thirdly, DCF is an extremely strong chemotherapy regimen with high toxicity. Improved DCF regimens with reduced toxicity have been reported (29,30,31), and the application of improved DCF regimens may be considered in future dual chemotherapy studies.

In summary, when using a reduced-dosage DCF regimen, patients with unresectable advanced gastric cancer could tolerate IP and IV dual chemotherapy. This regimen achieved acceptable survival results, including a TTP of five months and an MST of nine months. Future directions of study include using more efficient and less toxic chemotherapy drugs, such as oxaliplatin, capecitabine and S-1, to further improve the treatment efficacy and to reduce gastrointestinal side-effects, particularly abdominal pain.

## Figures and Tables

**Table I:** Patient characteristics.

Characteristic	Value
Gender, n	
Male	19
Female	13
Age, years	
Range	39–75
Median	65
Stage, n	
IIIB	6
IV	26
KPS	
Range	60–90
Median	70

KPS, Karnofsky performance status.

**Table II:** Hematological toxicities.

	Level I (6 cases)		Level II (26 cases)		Total (32 cases)	
			
	Cases, n	Cases, %	Cases, n	Cases, %	Cases, n	Cases, %
Leukopenia						
I–II	1	16.7	12	46.2	13	40.6
III–IV	5	83.3	14	53.8	19	59.4
Neutropenia						
I–II	1	16.7	10	38.5	11	34.4
III–IV	5	83.3	16	61.5	21	65.6
Febrile neutropenia	2	33.3	5	19.2	7	21.9
Anaemia						
I–II	2	33.3	12	46.2	14	43.8
III–IV	2	33.3	4	15.4	6	18.8
Thrombocytopenia						
I–II	1	16.7	9	34.6	10	31.3
III–IV	2	33.3	3	11.5	5	15.6

**Table III:** Non-hematological toxicity.

	Level I (6 cases)		Level II (26 cases)		Total (32 cases)	
			
	Cases, n	Cases, %	Cases, n	Cases, %	Cases, n	Cases, %
Abdominal pain						
I–II	2	33.3	18	69.2	20	62.5
III–IV	4	66.7	8	30.8	12	37.5
Anorexia						
I–II	2	33.3	13	50.0	15	46.9
III–IV	4	66.7	3	11.5	7	21.9
Fatigue						
I–II	3	50.0	20	76.9	23	71.9
III–IV	3	50.0	4	15.4	7	21.9
Nausea						
I–II	3	50.0	13	50.0	16	50.0
III–IV	3	50.0	4	15.4	7	21.9
Vomiting						
I–II	4	66.7	10	38.5	14	43.8
III–IV	2	33.3	2	7.7	4	12.5
Diarrhea						
I–II	1	16.7	9	34.6	10	31.3
III–IV	2	33.3	3	11.5	5	15.6
Abdominal distension						
I–II	3	50.0	14	53.8	17	53.1
III	2	33.3	4	15.4	6	18.8
Neurosensory						
I–II	1	16.7	4	15.4	5	15.6
III–IV	0	0.0	2	7.7	2	6.3
